# Supramolecular polymerization of sulfated dendritic peptide amphiphiles into multivalent L-selectin binders

**DOI:** 10.3762/bjoc.17.10

**Published:** 2021-01-12

**Authors:** David Straßburger, Svenja Herziger, Katharina Huth, Moritz Urschbach, Rainer Haag, Pol Besenius

**Affiliations:** 1 Department of Chemistry, Johannes Gutenberg University Mainz, Duesbergweg 10–14, 55128 Mainz, Germany; 2 Institute of Chemistry and Biochemistry, Freie Universität Berlin, Takustrasse 3, 14195 Berlin, Germany; 3 Research Center of Electron Microscopy, Freie Universität Berlin, Fabeckstr. 34a, 14195 Berlin

**Keywords:** L-selectin binders, multivalency, self-assembly in water, supramolecular polymers

## Abstract

The synthesis of a sulfate-modified dendritic peptide amphiphile and its self-assembly into one-dimensional rod-like architectures in aqueous medium is reported. The influence of the ionic strength on the supramolecular polymerization was probed via circular dichroism spectroscopy and cryogenic transmission electron microscopy. Physiological salt concentrations efficiently screen the charges of the dendritic building block equipped with eight sulfate groups and trigger the formation of rigid supramolecular polymers. Since multivalent sulfated supramolecular structures mimic naturally occurring L-selectin ligands, the corresponding affinity was evaluated using a competitive SPR binding assay and benchmarked to an ethylene glycol-decorated supramolecular polymer.

## Introduction

Deciphering the interaction of artificial molecular building blocks with biological components is a key element on the way to understanding and selectively manipulating biological systems. Throughout nature, these interactions occur in a multivalent fashion, allowing to overcome drawbacks of the limited strength of noncovalent bonds and to tune the selectivity at the same time [1–2]. The binding of viruses to the membrane of their host cells [3–5] as well as the recognition of carbohydrates by lectins [6–9] are only few of the numerous examples for multivalent protein–protein or protein–carbohydrate interactions that underline their pivotal role in biology. Mimicking polyvalency using synthetic systems has therefore become a growing field and the high degree of functionality renders polymers as a promising class of synthetic scaffolds [10–14].

Supramolecular polymers do provide additional features like a high degree of flexibility and excellent adaptivity, which are critical in biological interactions [15]. As water is the dominant solvent in biological systems, aqueous self-assembly turns out to be crucial in obtaining supramolecular polymers suitable for interactions with biological targets [16]. Peptide amphiphiles provide access to supramolecular structures in this competitive environment by taking advantage of nature’s versatile toolbox of noncovalent interactions [17–18]. By a careful design of the corresponding building blocks, extensive multilateral hydrogen bonds between the amino acid sequences of the oligopeptide backbone lead to secondary structures that direct the equilibrium to polymeric nano-scaled assemblies.

A well-studied receptor making use of multivalent interactions is the extracellular adhesion protein L-selectin. L-Selectin plays a critical role in inflammation processes by supporting the migration of leukocytes to inflammatory sites via adhesion to endothelial cells [19–21]. On a molecular level, a cationic binding site [22] promotes the binding of ligands exhibiting a high local negative charge density, such as sulfotyrosinated P-selectin glycoprotein ligand-1 (PSGL-1) [23] or heparin [24]. A versatile synthetic ligand that takes advantage of binding to cationic target sites, is dendritic polyglycerol sulfate [25] (dPGS), due to its functionalization with negatively charged sulfate groups. The binding behavior of dPGS to L-selectin has been thoroughly probed as dendrimer [26–27], conjugated to a polymer [28] or as amphiphilic adamantyl conjugates that are able to self-assemble on cyclodextrin vesicles [29].

Our group recently reported the successful application of functional supramolecular polymers in a biological context [30]. By decoration of the discotic peptide amphiphile monomers with dendritic mannose moieties, a specific cell targeting of macrophages and internalization in those antigen presenting cells has been achieved. As specific biological interactions strongly rely on the receptor–ligand interplays, we are interested in investigating the targeting of isolated receptors by supramolecular polymers built from peptide amphiphiles decorated with suitable ligand structures. The well-known L-selectin described above represents an excellent target that can be addressed by the multivalent presentation of sulfate groups. We therefore aim for the synthesis of sulfate-modified peptide amphiphiles to gain access to sulfated supramolecular polymers via self-assembly in water. The versatility of supramolecular structures modified with sulfate groups, and their capability to interact with biological components has been demonstrated recently [31–32]. In this work, we therefore coupled dPGS to *C*_2_-symmetrical discotic peptide amphiphiles using copper-catalyzed azide alkyne cycloaddition chemistry. The evaluation of the effect of sulfate modification on the self-assembly properties of the dendritic peptide amphiphiles were performed using circular dichroism (CD) spectroscopy and electron transmission microscopy (TEM) as well as cryogenic TEM. Finally, the binding affinity of the sulfated supramolecular polymers towards L-selectin has been evaluated using surface plasmon resonance (SPR) experiments.

## Results and Discussion

### Dendritic peptide amphiphile design and synthesis

**Scheme 1:**
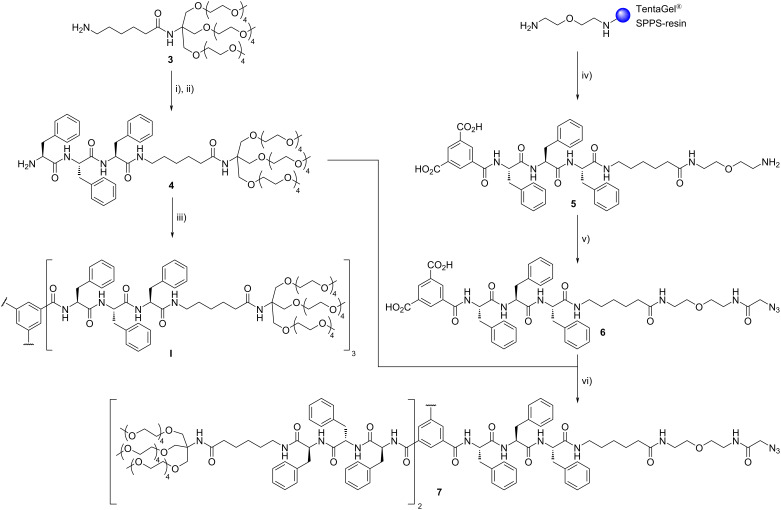
The synthesis of the *C*_3_-symmetrical tetraethylene glycol-decorated peptide amphiphile **I** and the azide-functionalized peptide amphiphile **7**: i) Boc-PhePhePhe-OH, HBTU, HOBt, DIPEA, DMF, 0 °C to rt, 15 h, 85%; ii) TFA/TIS/H_2_O 9.5:0.25:0.25 (v/v/v), rt, 1 h, 98%; iii) BTA, PyBOP, HOAt, NMM, DMF, 0 °C to rt, 15 h, 93%; iv) SPPS using HBTU, HOBt, DIPEA for coupling (HATU for BTA coupling), 20 vol % piperidine in DMF for Fmoc cleavage, DCM/TFA/TIS 20:20:1 (v/v/v) for final cleavage, 68%; v) azidoacetic acid NHS ester, NMM, DMF, rt, 4 h, 81%; vi) **4**, PyBOP, HOAt, NMM, DMF, 0 °C to rt, 18 h, 94%.

Two different peptide amphiphiles were synthesized, i.e., a non-sulfated, neutral amphiphile **I** as well as a dPGS-coupled, sulfated amphiphile **II**. Both amphiphiles were synthesized using a convergent and modular strategy as shown in Scheme 1. The Newkome-type [33] dendritic dodeca(ethylene glycol) moiety **3**, equipped with a 6-aminohexanoic acid spacer was synthesized as reported previously [34]. Using this building block in the subsequent HBTU-mediated amidation of Boc-protected tri-ʟ-phenylalanine, as β-sheet directing peptide sequence, afforded the peptide amphiphile in good yields. After deprotection of the *N*-terminus with TFA, molecule **4** was suitable for coupling to the branching unit to obtain the desired dendritic peptide amphiphiles. In case of the *C*_3_-symmetric amphiphile **I**, the readily available benzene-1,3,5-tricarboxylic acid (trimesic acid) was chosen as the branching unit. In the final amidation reaction, the efficient PyBOP-mediated coupling was carried out using an excess of the peptide amphiphile. Finally, the target compound **I** was purified by size exclusion chromatography.

In order to synthesize the sulfated, functionalized supramolecular building block **II**, we made use of the selective heterofunctionalization of trimesic acid. By replacing one of the solubilizing dodeca(ethylene glycol) moieties with an azide group, post-functionalization using a subsequent copper-catalyzed azide–alkyne cycloaddition reaction became accessible [35–36]. At the same time the other two unmodified side arms of the dendritic amphiphile make sure that the fidelity of the β-sheet motifs and directed supramolecular polymerization remains intact. Commonly this heterofunctionalization is achieved using esterification of trimesic acid and subsequent partial hydrolysis, however, this route suffers from difficult purification steps [37–38]. Here, it was conveniently achieved in one step via a solid-phase supported approach, using trimesic acid as capping reagent for the synthesized oligopeptide on the resin.

To maintain orthogonal functionalities a resin-bound diamine was deployed as starting point to start off the oligopeptide synthesis. A low loading of the resin (0.16 mmol/g) was important to prevent crosslinking during the reaction with trimesic acid. The coupling steps were performed using HBTU, HOBt, DIPEA in DMF with Fmoc derivatives of ʟ-phenylalanine and ε-aminohexanoic acid while the final capping was achieved using HATU, HOAt, DIPEA in NMP (Scheme 1). After cleavage from the resin the hetero-trifunctional peptide was purified by RP-HPLC and separated from impurities, like disubstituted byproduct, to finally afford **5** in 68% yield. Subsequently, an azide functionality was easily introduced using azidoacetic acid NHS ester to obtain compound **6**. In the next step, the solubilizing side-arm **4** was attached twice to the trimesic acid scaffold by PyBOP coupling in DMF and NMM as weak base. After subsequent size exclusion chromatography, the ready to use azide-functional monomer **7** was isolated in high purity.

**Scheme 2:**
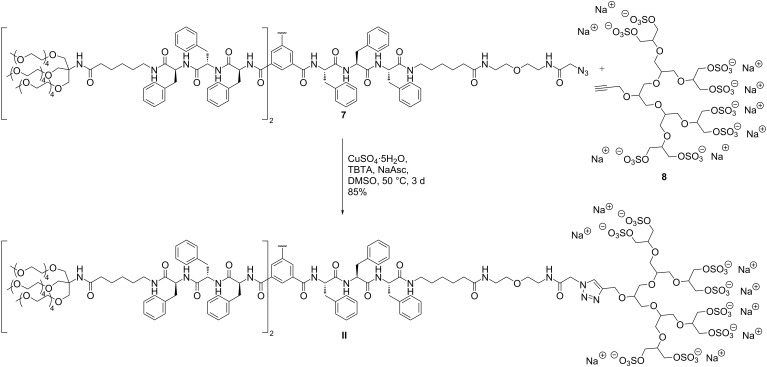
Synthesis of the sulfated peptide amphiphile **II** by copper-catalyzed azide–alkyne cyclization.

The peptide amphiphile was finally conjugated to the propargylated sulfated [G2] oligoglycerol dendron **8** via a copper-catalyzed azide–alkyne cycloaddition (Scheme 2). The reaction took place in degassed DMSO at 50 °C with CuSO_4_ pentahydrate, sodium ascorbate and tris(benzyltriazolylmethyl)amine (TBTA) as chelating species. HPLC-monitoring of the reaction showed a full conversion after three days and the crude mixture was subsequently purified by size exclusion chromatography to yield 85% of the oligosulfated monomer **II**.

### Characterization of the supramolecular polymers of **I** and **II**

**Figure 1:**
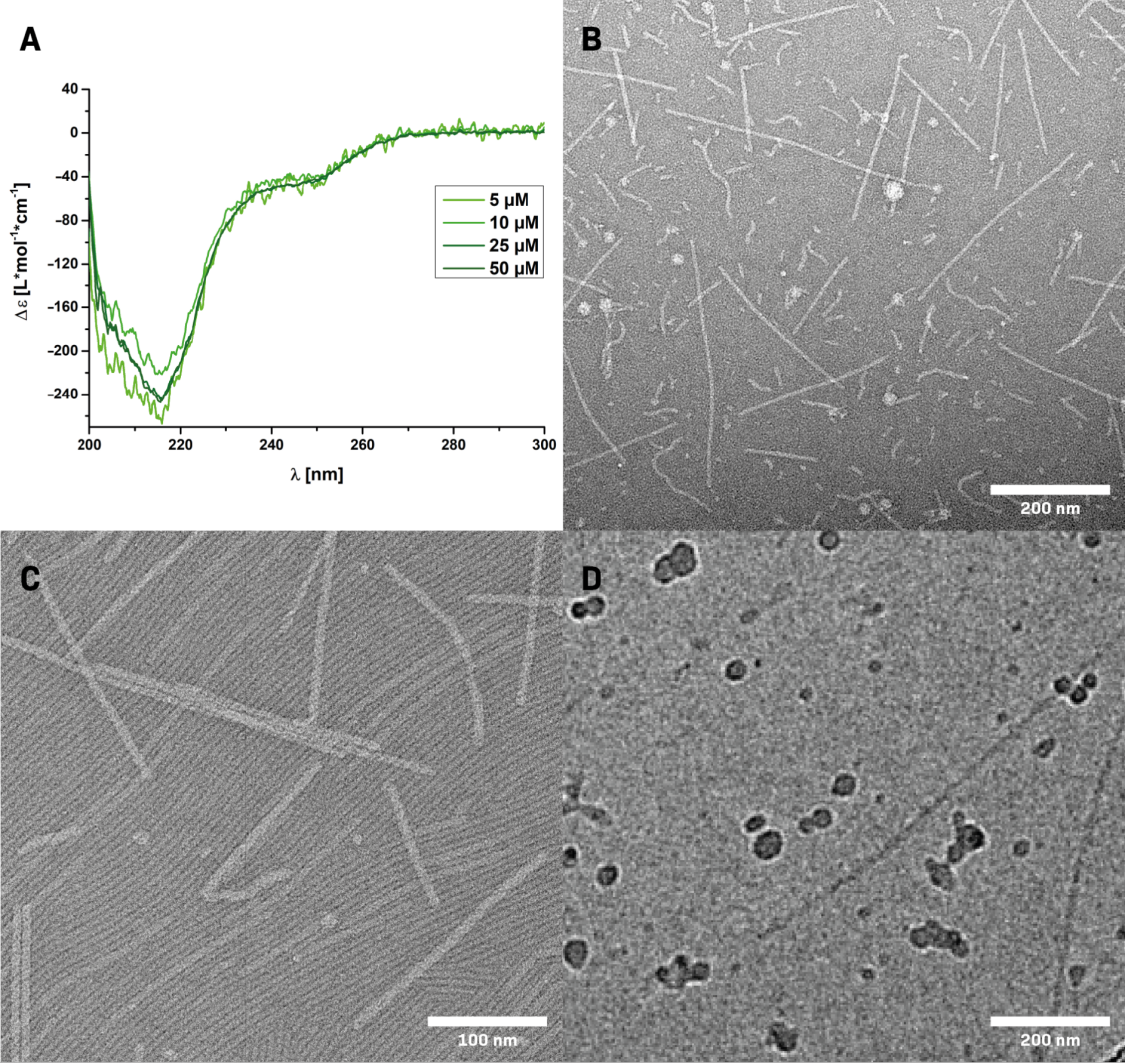
Analysis of the self-assembly behavior of **I** by A: CD-spectra of 5, 10, 25 or 50 µM aqueous solutions; B, and C: TEM micrographs of negatively stained 25 µM solutions in water; D: cryo-TEM micrograph of **I** (50 µM) in PBS (−/−) with 100 mM NaCl.

The self-assembly behavior of the peptide-based materials can be readily investigated using CD spectroscopy. The supramolecular polymerization strongly relies on the formation of secondary structure elements, leading to ordered domains that lead to characteristic CD band spectra. By probing aqueous solutions of **I** at different concentrations in H_2_O, a strongly negative CD band at around λ ≈ 220 nm became apparent (Figure 1A). In the past we have assigned this characteristic CD band to a β-sheet-dominated short-range ordering of oligopeptide monomers, which is stable over a wide concentration range [30,39–40].

The application of transmission electron microscopy (TEM) gave further insight into the morphology of the assemblies induced by the secondary structure formation (Figure 1). All samples for convential TEM imaging were prepared by placing 5 μL of the aqueous solution on a sample grid for 1 min, followed by the removal of the excess liquid using a filter paper and subsequent negative staining using a 2% uranyl acetate solution. By analyzing a 25 μM aqueous solution of **I**, long rigid anisotopic rod-like structures with lengths of 200–400 nm could be visualized (Figure 1B and 1C), in agreement with our previously reported findings [30,39]. These results confirm the high propensity of the branched nonaphenylalanine-derived molecules to form supramolecular polymers. The copresence of shorter, more flexible structures could be seen as well. Under higher magnification, a sheet like arrangement of densely packed and oriented rods could be revealed. Since the morphology of the aqueous supramolecular polymers observed in dry TEM can be influenced by the sample preparation, cryo-TEM experiments were furthermore performed in order to visualize the assemblies in a native state (Figure 1D and Figure S1 in Supporting Information File 1). The presence of straight nanorods of over 200 nm in length with a high degree of lateral order could be observed, thus corroborating the interpretations from dry TEM experiments.

**Figure 2:**
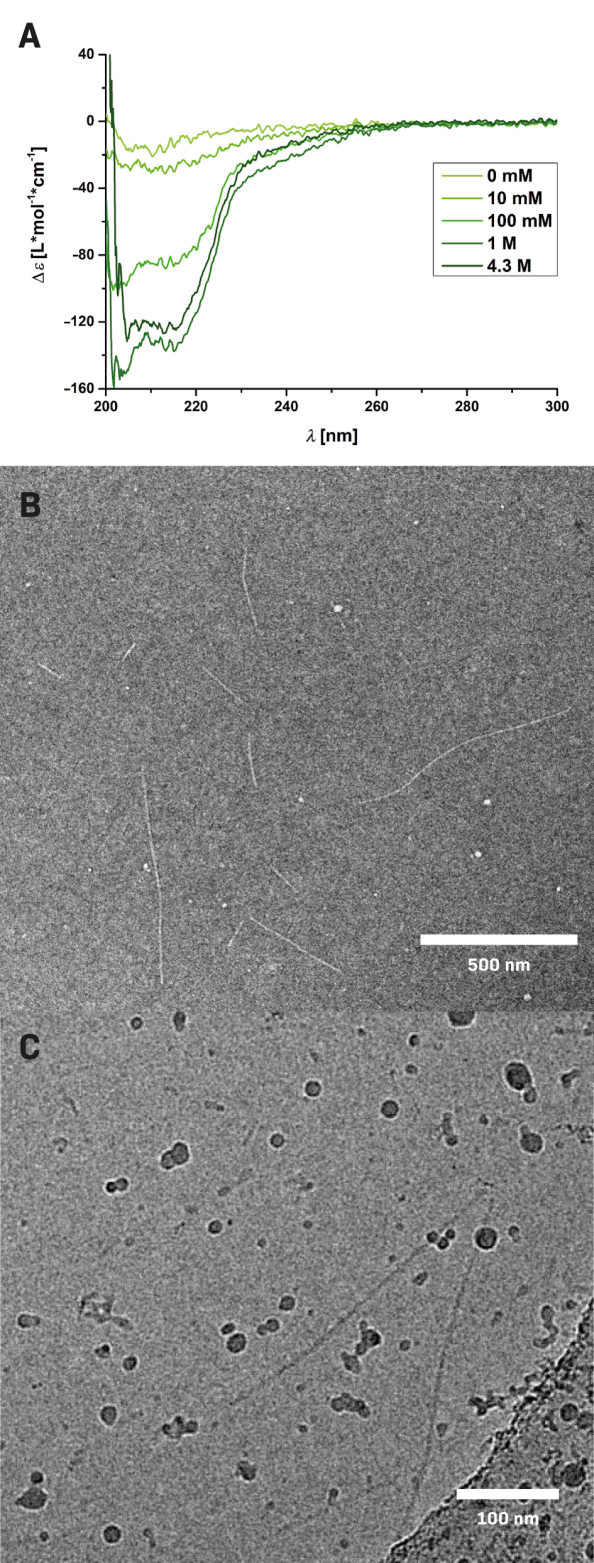
Analysis of the supramolecular polymerization of **II** by A: CD-spectra of a 25 µM solution in TRIS buffer (20 mM, pH 7.4) containing different concentrations of NaCl; B: TEM micrograph of a negatively stained 25 µM solution in TRIS buffer (20 mM, pH 7.4) containing 100 mM NaCl; C: Cryo-TEM micrograph of a 50 µM solutions in PBS buffer (−/−), pH 7.4 containing 100 mM NaCl.

Attaching functional groups to the dendritic peptide amphiphiles – and thus the supramolecular polymers – by exchanging one of the shielding dendritic tetra(ethylene glycol) dendrons is important with regard to potential applications. However, depending on the nature of the functional groups, the influence on the self-assembly behavior may not be neglected. In the present work, the installation of a sulfated oligoglycerol dendron introduces eight anionic charges to the periphery of the peptide amphiphile **II** that possibly counteracts the β-sheet-driven self-assembly due to electrostatic repulsion. CD spectroscopic experiments based on 25 µM or 50 µM solutions of **II** in 20 mM TRIS as well as PBS buffer support this notion (Figure 2A and Figure S2 in Supporting Information File 1). In pure 20 mM TRIS buffer (pH 7.4) the β-sheet characteristic negative CD band around λ ≈ 218 nm is weakened by more than a factor ten compared to the uncharged species **I**. However, in PBS buffer, providing a significantly higher salt concentration and physiological ionic strength, the corresponding CD signal from **II** is much more pronounced. We therefore decided to perform ionic strength-dependent titrations using a 25 µM solution of **II** in 20 mM TRIS at neutral pH. At >100 mM added NaCl, the characteristic β-sheet signature is restored due to charge screening of the peripheral sulfate groups (Figure 2A and Figure S3 in Supporting Information File 1). These results are in agreement with our previous investigations related to pH-switchable and ionic strength-responsive dendritic amphiphiles appended with dendritic carboxylic acid functionalities [40–41]. The physiological ionic strength thus efficiently screens the repulsive contribution of the sulfated dendrons and supports the application of the supramolecular polymers in biological applications.

In order to compare the morphology of the self-assembled sulfated compound **II** with the TEG-decorated compound **I**, TEM and cryo-TEM experiments were performed (Figure 2B and 2C). Based on the outcome from CD spectroscopy, we decided to carry out the TEM experiments in solutions of neutral pH and physiological ionic strength, to efficiently screen the repulsive charges at the surface of the sulfated dendritic peptide amphiphile **II**. For the dry TEM experiments we preferred 10 mM or 20 mM TRIS buffer containing 100 mM NaCl, over the phosphate buffer in PBS, since TRIS is known to avoid artefacts due to phosphate salts [42–43]. Cryo-TEM experiments were performed using solutions of **II** in PBS (−/−, containing 100 mM NaCl). All TEM and cryo-TEM micrographs clearly demonstrated the formation of long rigid rods in the range of several hundred nanometers (Figure 2B,C, and Figure S4 in Supporting Information File 1). These results thus confirm our conclusions from CD spectroscopy and prove an ionic strength-dependent β-sheet ordering in neutral or anionic sulfated dendritic peptides. Under physiological conditions, supramolecular polymer formation with a length of several hundred nanometers is observed at monomer concentrations as low as 25 µM.

### Evaluation of the L-selectin binding efficacy

**Figure 3:**
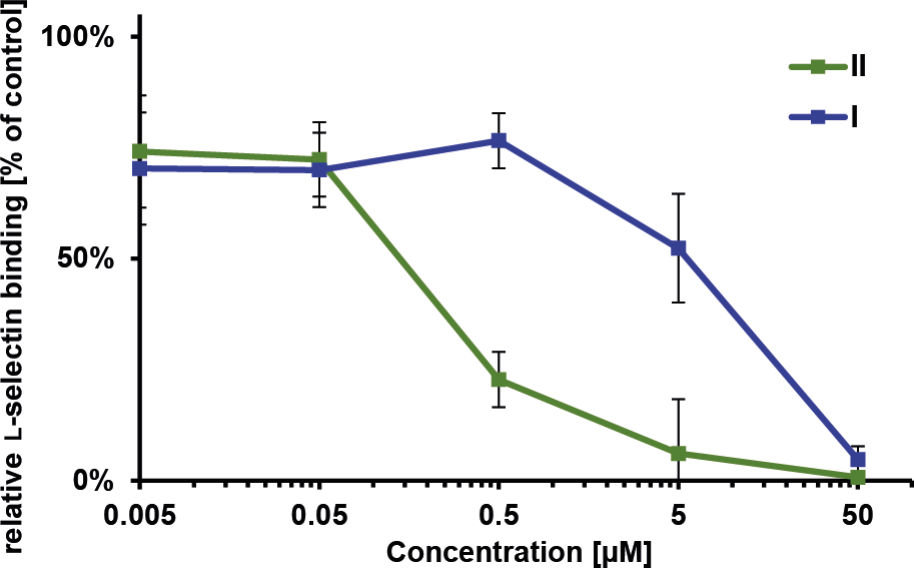
Concentration-dependent relative L-selectin binding of the supramolecular polymers **I** and **II** in HEPES buffer (0.020 M, pH 7.4, with 0.150 M NaCl and 0.001 M CaCl_2_) measured by SPR. L-Selectin-coated nanoparticles were preincubated with solutions of the supramolecular polymers. Their affinity towards multimerized artificial ligands composed of sulfated tyrosine and sialyl-Lewis^X^, immobilized on an SPR-chip was probed. The binding signal is plotted relative to a sample without preincubation (100%).

The L-selectin binding behavior of the sulfated supramolecular polymer resulting from **II** was assessed using a competitive surface plasmon resonance (SPR) binding assay, whereby neutral supramolecular polymers of **I** were used as control experiment. L-Selectin-coated gold nanoparticles were incubated with analyte solutions of different concentrations and subsequently probed on an SPR-chip modified with immobilized sulfated tyrosine and sialyl-Lewis^X^ as model ligands. The resulting signal is referenced to a control experiment lacking the preincubation of L-selectin particles. Figure 3 summarizes the resulting dose–response curves.

The sulfated supramolecular assemblies resulting from **II** bound with an IC_50_ value of 250 nM which is 20-fold lower compared to the unfunctionalized tetra(ethylene glycol)-decorated supramolecular polymers **I** (IC_50_ = 5 µM). Further, the affinity is significantly higher compared to naturally binding unfractionated heparin (15 kDa, IC_50_ = 12 µM) or artificial polyglycerol sulfate of similar molecular weight and number of sulfate groups (dPG_3kDa_S11: IC_50_ = 17 µM; dPG_6kDa_S8: IC_50_ = 50 µM) [26]. However, the densely sulfated dendritic polyglycerol of similar molecular weight is able to bind in the low nanomolar range suggesting that a high density of functional groups is a key parameter [26]. These results show that the multivalent presentation of sulfate moieties on the surface of one-dimensional anisotropic supramolecular polymers noticeably enhances their affinity towards surface immobilized L-selectin. However, the binding affinity of the unmodified supramolecular polymer **I** was unexpectedly high. This may be attributed to an unspecific adhesion due to hydrophobic patches, since the shielding oligo(ethylene glycol) units are relatively short.

## Conclusion

In this work, we presented the charge-regulated β-sheet-driven supramolecular polymerization of a sulfated peptide amphiphile and its neutral analog, as well as their L-selectin binding behavior. A branched nonaphenylalanine-based peptide amphiphile was synthesized, which carries two solubilizing side arms with tetra(ethylene glycol) chains and one arm with an azide functional group. This modular building block was obtained using a high yielding novel solid phase approach and will be further used for dendritic peptide amphiphile heterofunctionalization. By taking advantage of copper-catalyzed azide–alkyne cycloaddition chemistry, a highly soluble sulfated nonaphenylalanine peptide amphiphile was prepared via this route. The spectroscopic and microscopic investigations of the aqueous self-assembly behavior of the sulfated peptide amphiphile revealed an ionic strength-dependent formation of one-dimensional anisotropic supramolecular polymers, whereas the non-ionic tetra(ethylene glycol)-decorated analog showed supramolecular polymerization without the addition of salts. Finally, SPR experiments provided evidence for a high affinity binding of the multivalent oligosulfate groups on the periphery of the supramolecular polymers towards surface-immobilized L-selectin. Our studies suggest that supramolecular polymers will be an applicable platform to prepare and evaluate anti-inflammatory materials using multicomponent and multifunctional supramolecular subunits.

## Supporting Information

File 1 Experimental procedures, materials and methods, detailed synthetic procedures and the characterization of all molecules.
