# Supplementary material for: Supramolecular polymerization of sulfated dendritic peptide amphiphiles into multivalent L-selectin binders
Source: Beilstein J Org Chem. 2021 Jan 12;17:97–104. doi: 10.3762/bjoc.17.10 (PMC7814183; doi:10.3762/bjoc.17.10)
Supplement: File 1 — Experimental procedures, materials and methods, detailed synthetic procedures and the characterization of all molecules. [file Beilstein_J_Org_Chem-17-97-s001.pdf]

BEILSTEIN JOURNAL OF ORGANIC CHEMISTRY

Supporting Information

for

Supramolecular polymerization of sulfated dendritic peptide amphiphiles into multivalent L-selectin binders

David Straßburger, Svenja Herziger, Katharina Huth, Moritz Urschbach, Rainer Haag and Pol Besenius

Beilstein J. Org. Chem. **2021,** *17,* 97–104. doi:10.3762/bjoc.17.10

Experimental procedures, materials and methods, detailed synthetic procedures and the characterization of all molecules

License and Terms: This is a supporting information file under the terms of the Creative Commons Attribution License (https://creativecommons.org/licenses/by/4.0). Please note that the reuse, redistribution and reproduction in particular requires that the author(s) and source are credited and that individual graphics may be subject to special legal provisions.
The license is subject to the *Beilstein Journal of Organic Chemistry* terms and conditions: (https://www.beilstein-journals.org/bjoc/terms)

1 Supplementary figures

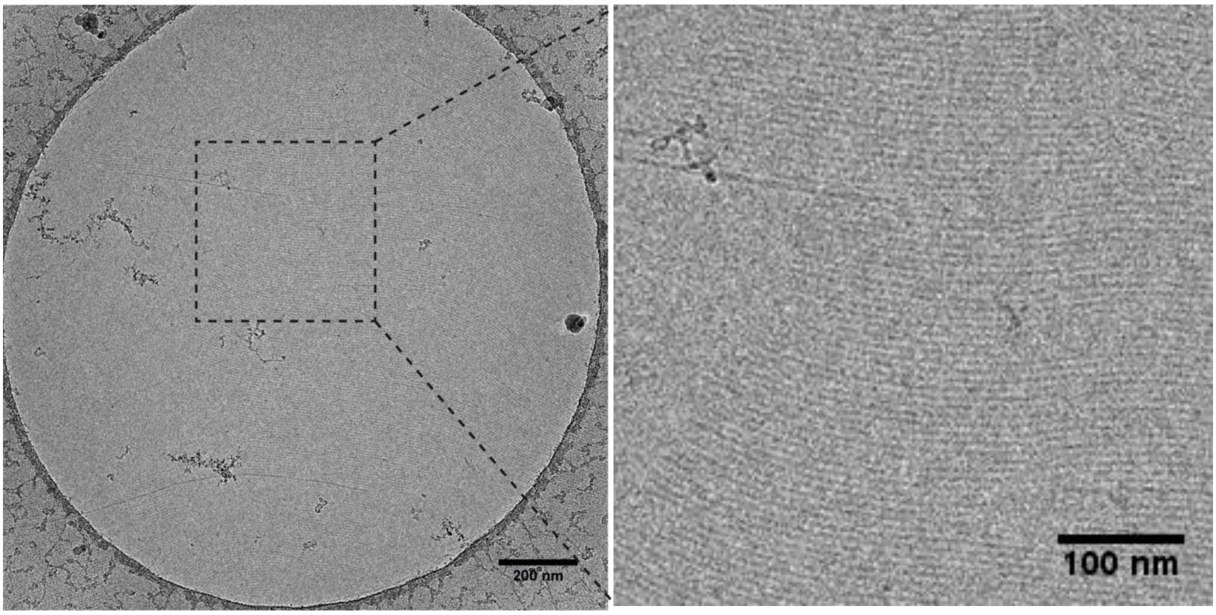

Figure S1: Cryo-TEM micrograph of **I** (50 µM) in PBS (-/-) with 100 mM NaCl and zoom-in (right).

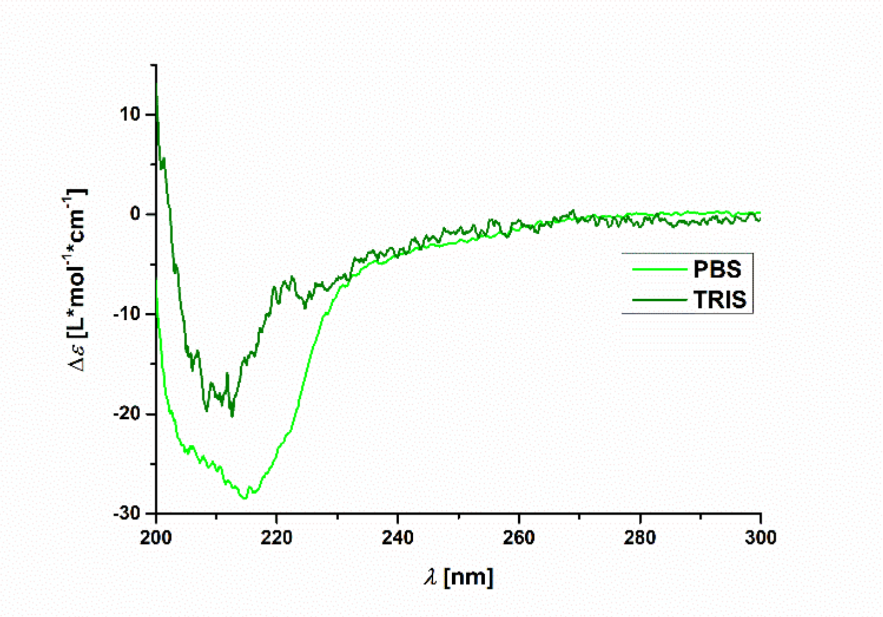

Figure S2: CD spectra of **II**; both samples prepared as 50 µM solutions in PBS or Tris buffer (20 mM) at pH 7.4 at 20 °C.

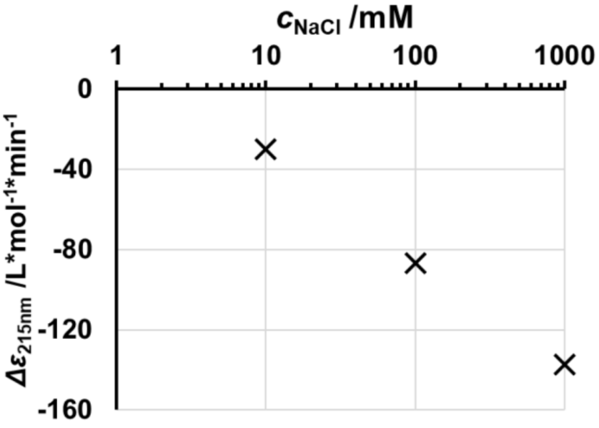

Figure S3: Molar circular dichroism of a 25 µM solution of **II** in 20 mM Tris (pH 7.4), plotted as a function of the NaCl concentration.

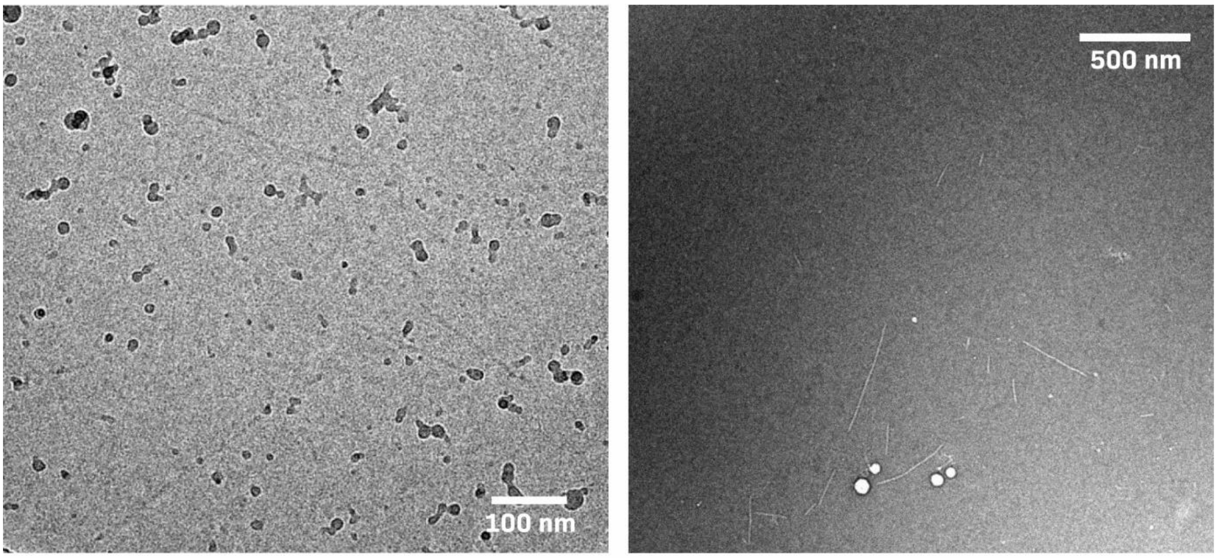

Figure S4: Additional cryo-TEM micrograph of a 50 µM solution of compound **II** in PBS buffer (-/-) pH 7.4 containing 100 mM NaCl (left) and the TEM micrograph of a negatively stained 25 µM solution of compound **II** in TRIS buffer (20 mM, pH 7.4) containing 100 mM NaCl.

2 General considerations

Solvents and reagents

Unless stated otherwise, all solvents and reagents were obtained from commercial sources in at least pro analysi (p.a.) quality and were used without further purification. The absolutation of solvents was performed according to literature-known procedures [S1]. Ultrapure water was obtained from an Elga PURELAB flex 4 system by Veolia (Paris, France). The propargylated [G2]-dendron was synthesized in accordance to literature-known procedures [S2].

Reaction conditions

Air- and moisture-sensitive reactions were carried out under argon atmosphere with common Schlenk techniques and dry solvents. For this purpose, all glassware were previously oven-dried or dried via a heat gun in vacuo at least three times. In addition, all necessary solvents and liquid starting materials were added via a septum and with disposable syringes, which were previously flushed with argon. Solids were added under a continuous counterflow of argon.

Chromatography

All flash-chromatographic purifications were carried out using silica gel with an average particle size of 35–70 µm and a pore size of 60 Å by Acros Organics (Geel, Belgium). A nitrogen pressure of 0.3–0.5 bar was applied. The eluents were freshly prepared of pro analysi grade solvents or distilled technical grade solvents. The analysis of the collected fractions was performed via TLC. The TLC-analyses were carried out on silica-coated aluminum sheets 60 F254 by Merck KGaA (Darmstadt, Germany) with fluorescence indicator. The analytes were detected by the following methods: UV absorption at a wavelength of 254 nm; vanillin-stain (solution prepared of 100 mL methanol with 1.0 g vanillin, 12 mL acetic acid, and 4mL conc. sulfuric acid); ninhydrin stain (solution prepared of 1.5 g ninhydrin in 500 mL methanol and 15 mL acetic acid); $KMnO_4$ stain (solution prepared of 6 g $KMnO_4$, 40 g K_2CO_3 and 13 mg NaOH in 600 mL H_2O. In case of staining agents, the plate is immersed shortly and the color reaction subsequently proceeds upon heating.

NMR spectroscopy

All ^{1}H and ^{13}C NMR spectra were recorded on a Bruker Avance II 400 spectrometer equipped with a 5 mm broadband observe probehead (z-gradient) using standard Bruker release pulse sequences (^{1}H NMR: 400 MHz, ^{13}C NMR: 100.7 MHz) or a Bruker Avance III 600 spectrometer

equipped with a 5 mm cryogenic triple-band inverse probehead (z-gradient) using standard Bruker release pulse sequences (^{1}H NMR: 600 MHz, ^{13}C NMR: 150.9 MHz) both by Bruker (Rheinstätten, Germany). The samples were dissolved in deuterated solvents obtained from Deutero (Kastellaun, Germany). The chemical shifts (δ/ppm) are referenced to the residual proton signal of the deuterated solvent and are reported in parts per million (ppm) relative to tetramethylsilane (δ = 0.00 ppm). Coupling constants (*J*) are calculated in hertz (Hz) and the spin multiplicity of the signals is reported using following abbreviations: bs = broad singlet, s = singlet, d = doublet, t = triplet, q = quartet and m = multiplet, as well as appropriate combinations of these. The following abbreviations for substructures are used in the course of the proton assignments: Q = quinoline, XDA = xylylenediamine, IQ = imidazoquinoline, MI = maleimide, Bu = butyl. The spectra were processed and analyzed using MestReNova 14.1.0 by MESTRELAB RESEARCH (Santiago de Compostela, Spain).

Mass spectrometry

High resolution electrospray ionization mass spectra (ESI–HRMS) were recorded on an Agilent 6545 Q-TOF mass spectrometer (Agilent Technologies, Santa Clara, USA) with a LockSpray interface. Alternatively, ionization using field desorption was performed on a MAT 95 by Finnigan (San Jose, USA). MALDI samples were measured on an Autoflex maX MALDI-TOF/TOF device by Bruker. The corresponding matrices used are stated in the synthetic procedures.

CD

All circular dichroism spectra were recorded on a J-815 spectrometer from JASCO (Tokyo, Japan) and processed with Spectra Manager Version 2.12.00. The samples were measured using 110-QS cuvettes made of SUPRASIL® quartz glass by Hellma (Mühlheim, Germany) with a path length of 2 mm at 293°K, if not stated otherwise. The spectra were plotted in MS Excel® 365. For each solvent a blank spectrum was measured and subtracted from the raw data.

TEM

The transmission electron microscopy (TEM) images were recorded on a Tecnai T12 by FEI (Hillsboro, USA), which was equipped with a LaB6 cathode operating at 120 kV. The electron micrographs were recorded with a 4k × 4k CMOS by TVIPS (Oslo, Norway). The copper grids CF300-CU with a 3–4 nm thick carbon film from Electron Microscopy Sciences (Hatfield, USA) were glow discharged prior to use. For each analysis, 5 µL (10–100 µM aqueous solution) of the test item was adsorbed on the grid for 2 min. The grids were then stained for 1 min with 2.0 wt % uranyl acetate from Polysciences (Warminster, USA). The generated droplet was

removed with a Whatman® grade 4 filter paper tip from GE Healthcare Bio Sciences (Uppsalla, Sweden).

Cryo TEM

Droplets of the sample solution (5 µL) were applied on hydrophilized holey carbon filmed grids (Quantifoil R1/4) at room temperature. Hydrophilization was achieved beforehand by 60 s glow discharging in a BALTEC MED 020 (BALTEC, Liechtenstein) at 8.5 mA. The vitrified samples were then prepared using a VitrobotTM Mark IV (Thermo Fisher Scientific) at room temperature and a blotting time of 3 s.

The vitrified samples were transferred under liquid nitrogen temperature into a Talos Arctica 200 kV transmission electron microscope (Thermo Fisher Scientific), using the microscope's autoloader protocol. The micrographs were recorded under low-dose conditions with a primary magnification of 28000x and an acceleration voltage of 200 kV. The images were recorded by a Falcon III direct electron detector at full 4k resolution resulting in a pixel size of 0.373 nm/pixel.

L-Selectin binding assay

The L-selectin binding assay was performed in a similar manner as described in [S3]. The binding of soluble L-selectin binders was studied via a competitive SPR-based inhibition assay performed on a BIAcore X device (GE Healthcare, Freiburg, Germany) as previously explained in detail [S4-S6]. In brief, the assay relies on the L-selectin binding to an artificial ligand, which is composed of sulfated tyrosine and the tetrasaccharide sialyl Lewis X multimerized on a polyacrylamide backbone and attached via biotin to a streptavidin-coated gold chip (GE Healthcare, Freiburg, Germany). Protein A-coated gold nanoparticles (15 nm, Aurion, Wageningen, Netherlands) were loaded with an L-selectin/Fc chimera (R&D Systems, Minneapolis, USA), and the binding signal to the artificial ligand was recorded. Samples without inhibitors were set as a 100% binding control. In turn, binding signals of the L-selectin-loaded gold nanoparticles preincubated with different concentrations of the potential inhibitors were taken, which yielded the respective dose–response curves. The IC50 values were determined by fitting the relative binding response data with a sigmoidal dose–response curve.

3 Synthetic procedures

O-Azidoacetyl-*N*-hydroxysuccinimide, N_3AcNHS (**S1**)

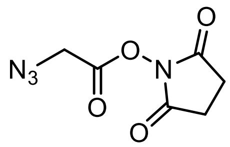

Azidoacetic acid (2.55 g, 2.52 mmol) was dissolved in 20 mL THF and cooled to 0 °C. Subsequently, NHS (2.88 g, 25 mmol, 1.0 equiv) was added in small portions. A solution of DCC (5.16 g, 25 mmol, 1.0 equiv) in 15 mL THF was added slowly. The reaction mixture was brought to room temperature and stirred for 4 h. The resulting suspension was filtered at 0 °C several times until no more urea was formed. The filtrate was diluted with 50 mL Et_2O and allowed to stand for 12 h at 3 °C. The precipitated product was filtered and washed with cold Et_2O.

Yield: 2.56 g (12.9 mmol, 51%) amorphous colorless solid.

$C_6H_6N_4O_4$ (M = 198.1380 g/mol, M_{mi} = 198.0389 u).

^{1}H NMR (400 MHz, DMSO-d_6, 296 K) δ /ppm = 4.71 (s, 2H, CH_2α), 2.84 (s, 4H, 2×CH_2NHS).

ε-Azidohexanoic acid (**S2**)

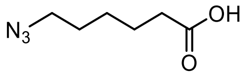

6-Bromohexanoic acid (4.08 g, 20.1 mmol, 1.0 equiv) was dissolved in 20 mL DMF and dry NaN_3 (4.08 g, 60.3 mmol, 3.0 equiv) was added. The slurry was stirred for 4 h at 40 °C. The whole reaction mixture was directly subjected to FC on silica (EA).

Yield: 2.40 g (15.3 mmol, 76%)

$C_6H_{11}N_3O_2$ (M = 157.1730 g/mol, M_{mi} = 157.0851 u).

TOF-MS (ESI, neg.) m/z: 156.0780 $[M-H]^-$ (calc. 156.0779).

^{1}H NMR (400 MHz, DMSO-d_6, 296 K) δ /ppm = 12.0 (s, 1H, CO_2H), 3.31 (t, 2H, $J_{ε,δ}$ = 6.9 Hz, $Ahx_ε$), 2.20 (t, 2H, $J_{α,β}$ = 7.3 Hz, $Ahx^α$), 1.59–1.45 (m, 4H, $Ahx^β$, $Ahx^δ$), 1.38–1.26 (m, 2H, $Ahx^γ$).

N$_3$AhxTRIS, **S3**

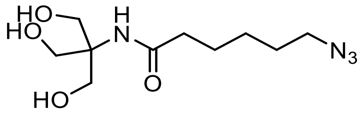

6-Azidohexanoic acid (2.26 g, 14.3 mmol, 1.1 equiv) was dissolved in 130 mL abs. EtOH. Subsequently, TRIS (1.57 g, 13.0 mmol, 1.0 equiv) and EEDQ (3.86 g, 15.6 mmol, 1.2 equiv) were added and the resulting suspension was refluxed for 20 h. Then, the alcohol was removed in vacuo and the remaining viscous oil was subjected to FC on silica (EA/MeOH + 0.1 vol % 7N NH_3 in MeOH, EA→4:1) to give the title compound.

Yield: 3.25 g (12.5 mmol, 87%) colorless viscous oil.

$C_{10}H_{20}N_4O_4$ (M = 260.2940 g/mol, M_{mi} = 260.1485 u).

TOF-MS: (ESI, pos.) m/z: 283.1368 $[M+Na]^+$ (calc. 283.1377).

^{1}H NMR: (400 MHz, DMSO-d_6, 296 K) δ /ppm = 7.12 (s, 1H, N*H*), 4.75 (d, 3H, $J_{OH,CH2}$ = 5.8 Hz, OH), 3.51 (d, $J_{CH2,OH}$ = 5.8 Hz, 3×CH_2^{TRIS}), 3.31 (q, $J_{\varepsilon,\delta}$ = 6.9 Hz, 2H, Ahx$^{\varepsilon}$), 2.14 (t, $J_{\alpha,\beta}$ = 7.3 Hz, 2H, Ahx$^{\alpha}$), 1.58–1.43 (m, 4H, Ahx$^{\beta}$, Ahx$^{\delta}$), 1.35–1.22 (m, 2H, Ahx$^{\gamma}$).

N$_3$AhxTRIS(EG$_4$OMe)$_3$, **S4**

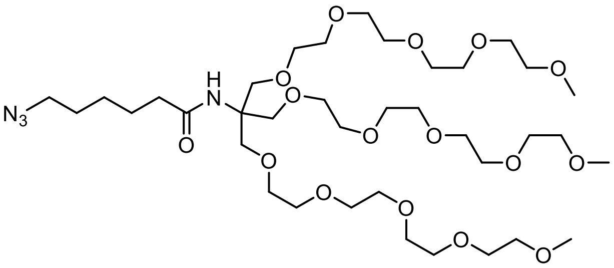

Alcohol **S3** (1.00 g, 3.84 mmol, 1.0 equiv) was dissolved in 20 mL distilled THF and cooled to 0 °C. Then, a NaH dispersion (57 wt %, 34 mg, 12.7 mmol, 3.3 equiv) was added in small portions and the resulting slurry was stirred for 15 min under argon atmosphere. Tetraethylene glycol monomethyl ether tosylate (6.062 g, 16.7 mmol, 4.3 equiv) was dissolved in 10 mL of distilled THF and added over 10 min. The reaction mixture was brought to room temperature and after 1 h of stirring and further addition of 30 mL anhydrous THF the mixture was heated to 40 °C. After 22 h the slurry was cooled to room temperature and 10 mL MeOH were added. The resulting solution was neutralized with AcOH. The solvents were removed in vacuo and the residue was taken up in EA and subsequently adsorbed on silica for purification via FC (EA/MeOH 20:1→4:1).

Yield: 2.48 (2.98 mmol, 78%) colorless viscous oil.

$C_{37}H_{74}N_4O_{16}$ (M = 831.0110 g/mol, M_{mi} = 830.5100 u).

TOF-MS (ESI, pos.) m/z: 853.4990 $[M+Na]^+$ (calc. 853.4998).

^{1}H NMR (400 MHz, DMSO-d_6, 296 K, COSY, HSQC, HMBC) δ /ppm = 7.02 (s, 1H, N*H*), 3.59 (s, 3×CH_2^{TRIS}), 3.55–3.46 (m, 42H, 21×CH_2-O), 3.45–3.40 (m, 6H, 3×CH_2-OMe), 3.24 (s, 9H, 3×CH_3-O), 3.30 (q, 2H, $J_{ε,δ}$ = 6.9 Hz, Ahx^ε), 2.06 (t, 2H, $J_{α,β}$ = 7.3 Hz, Ahx^α), 1.58–1.40 (m, 4H, Ahx^β, Ahx^δ), 1.34–1.21 (m, 2H, Ahx^γ).

N_3AhxTRIS(EG_4OMe)$_3$, S5

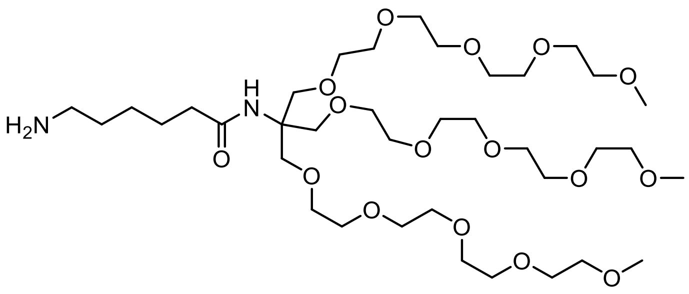

N_3AhxTRIS(EG_4OMe)$_3$ (**S4**, 859 mg, 1.03 mmol) was dissolved in 30 mL MeOH and Pd/C (60 mg, 10 wt %) was added under inert gas. A hydrogen atmosphere was established and the reaction mixture was vigorously stirred for 26 h. The reaction mixture was filtered over a Celite® pad and the solvent removed in vacuo to yield the product **S5**.

Yield: 760 mg (0.94 mmol, 92%) colorless viscous oil.

$C_{37}H_{76}N_2O_{16}$ (M = 805.0130 g/mol, M_{mi} = 804.5195 u).

TOF-MS (ESI, pos.) m/z: 805.5243 $[M+H]^+$ (calc. 805.5268), 827.5085 $[M+Na]^+$ (calc. 827.5087).

^{1}H NMR (400 MHz, DMSO-d_6, 296 K, COSY, HSQC, HMBC) δ /ppm = 7.02 (s, 1H, N*H*), 3.60 (s, 3×CH_2^{TRIS}), 3.54–3.46 (m, 42H, 21×CH_2-O), 3.45–3.41 (m, 6H, 3×CH_2-OMe), 3.24 (s, 9H, 3×CH_3-O), 2.58 (q, 2H, $J_{ε,δ}$ = 7.2 Hz, Ahx^ε), 2.05 (t, 2H, $J_{α,β}$ = 7.3 Hz, Ahx^α), 1.50–1.32 (m, 4H, Ahx^β, Ahx^δ), 1.31–1.19 (m, 2H, Ahx^γ).

[*N*-(*tert*-Butoxycarbonyl)]-L-phenylalanyl-L-phenylalanyl-L-phenylalanyl-{6-aminohexanoyl-*N*-methyl-[tri(2,5,8,11,14-pentaoxa-pentadecyl)]} amide, $BocF_3AhxTRIS(EG_4OMe)_3$ (**S6**)

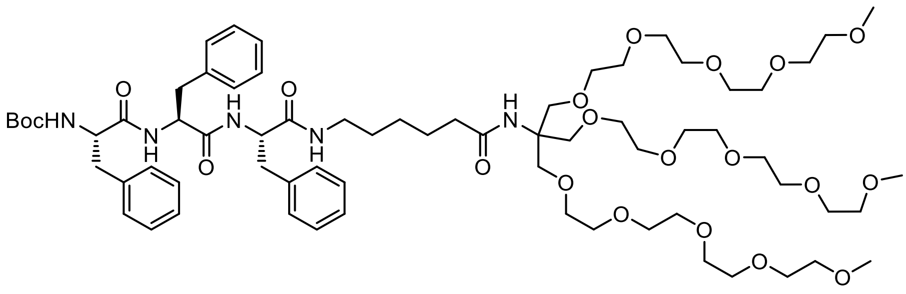

$BocF_3$ (263 mg, 0.47 mmol, 1.3 equiv), HBTU (178 mg, 0.47 mmol, 1.3 equiv), and HOBt (72 mg, 0.47 mmol, 1.3 equiv) were dissolved in DMF (2.5 mL) and after 10 min of cooling to 0 °C, DIPEA (63 µL, 0.36 mmol, 1.0 equiv) was added. The mixture was stirred for 10 min before it was introduced into a solution of $AhxTRIS(EG_4OMe)_3$ (300 mg, 0.36 mmol, 1.0 equiv) in DMF (0.5 mL). The cold reaction mixture was stirred for 15 min before two more equivalents of DIPEA (126 µL, 0.72 mmol, 2.0 equiv) were added. After another 15 min of stirring at 0 °C, the solution was warmed to room temperature and stirred for 15 h. The solution was quenched with H_2O and concentrated in vacuo. The residual oil was subjected to SEC on LH-20 (MeOH).

Yield: 423 mg (314 µmol, 85%) colorless oil.

$C_{69}H_{111}N_5O_{21}$ (M = 1346.6610 g/mol, M_{mi} = 1345.7772 u).

$\boldsymbol{R_F}$ = 0.48 ($CHCl_3$/MeOH, 10:1).

TOF-MS (ESI, pos.) m/z: 1368.7657 $[M+Na]^+$ (calc. 1368.7670).

^{1}H NMR (400 MHz, DMSO-d_6, 296 K) δ /ppm = 8.21 (d, 1H, $J_{NH,\alpha}$ = 8.2 Hz, NH^F), 7.89 (d, 1H, $J_{NH,\alpha}$ = 8.1 Hz, NH^F), 7.816 (t, 1H, $J_{NH,\varepsilon}$ = 5.6 Hz, NH^{Ahx}), 7.29–7.10 (m, 15H, 15×$F^{\delta,\varepsilon,\zeta}$), 7.00 (s, 1H, N$H^{TRIS}$), 6.9 (d, 1H, $J_{NH,\alpha}$ = 8.7 Hz, NH^{Boc}), 4.55 (td, 1H, $J_{\alpha,NH}$ = 8.3 Hz, $J_{\alpha,\beta}$ = 4.9 Hz, F^α), 4.46 (td, 1H, $J_{\alpha,NH}$ = 8.1 Hz, $J_{\alpha,\beta}$ = 6.1 Hz, F^α), 4.02 (td, 1H, $J_{\alpha,\beta1}$ = 9.4 Hz, $J_{\alpha,NH}$ = 8.7 Hz, $J_{\alpha,\beta2}$ = 3.9 Hz, $F^{\alpha1}$), 3.60 (sb, 6H, CH_2^{TRIS}), 3.54–3.39 (m, 42H, 21×CH_2O), 3.33 (s, 6H, 3×CH_2O), 3.23 (s, 9H, OCH_3), 3.17 (m, 3H, 3×F^β), 3.07–2.74 (m, 8H, Ahx^ε, 3×F^β), 2.03 (t, 2H, $J_{\alpha,\beta}$ = 7.5 Hz, Ahx^α), 1.41 (p, 2H, $J_{\beta,\alpha}$ = $J_{\beta,\gamma}$ = 7.6 Hz, Ahx^β), 1.34–1.21 (m, 11H, CH_3^{Boc}, Ahx^δ), 1.20–1.11 (m, 2H, Ahx^γ).

$F_3AhxTRIS(EG_4OMe)_4$, S7

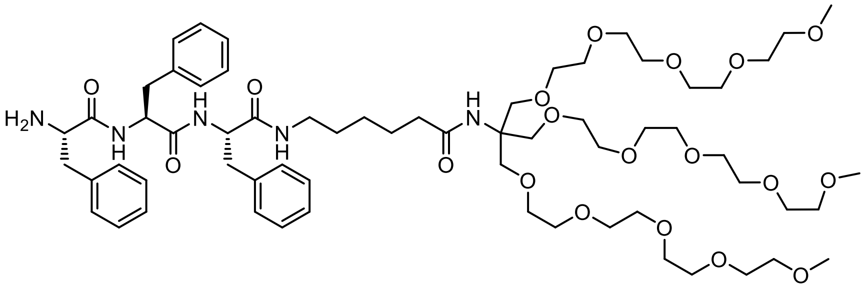

$BocF_3AhxTRIS(EG_4OMe)_3$ (420 mg, 312 µmol) was dissolved in a mixture of TFA/TIS/H_2O 9.5:0.25:0.25 (10 mL) and agitated for 1 h at room temperature. The solvents were removed in vacuo and the mixture was co-distilled with toluene (3 × 30 mL). The remaining oil was subjected to SEC (LH-20, MeOH + 0.1 vol % 7 N NH_3) followed by RP-MPLC (method A). The title compound was further converted into the hydrochloride.

Yield: 381 mg (305 µmol, 98%) colorless gel.

$C_{64}H_{103}N_5O_{19}$ (M = 1246.5440 g/mol, M_{mi} = 1245.7247 u).

$\boldsymbol{R}_F$ = 0.35 ($CHCl_3$/MeOH, 10:1).

TOF-MS (ESI, pos.) m/z: 1246.7320 $[M+H]^+$ (calc. 1246.7325).

^{1}H NMR (400 MHz, DMSO-d_6, 296 K, COSY, HSQC) δ /ppm = 9.01 (d, 1H, $J_{NH,\alpha}$ = 8.1 Hz, NH^F), 8.48 (d, 1H, $J_{NH,\alpha}$ = 8.3 Hz, NH^F), 8.26 (sb, 3H, NH_3), 7.96 (t, 1H, $J_{NH,\varepsilon}$ = 5.6 Hz, NHAhx), 7.33–7.12 (m, 15H, 15×$F^{\delta,\varepsilon,\zeta}$), 7.03 (s, 1H, N$H^{TRIS}$), 4.56 (td, 1H, $J_{\alpha,NH}$ = 8.4 Hz, $J_{\alpha,\beta}$ = 5.1 Hz, F^α), 4.49 (td, 1H, $J_{\alpha,NH}$ = 8.2 Hz, $J_{\alpha,\beta}$ = 6.2 Hz, F^α), 4.02 (q, 1H, $J_{\alpha,\beta}$ = 5.9 Hz, $F^{\alpha1}$), 3.66–3.38 (m, 54H, CH_2^{TRIS} {3.60}, 24×CH_2O), 3.23 (s, 9H, OCH_3), 3.17 (m, 3H, 3×F^β), 3.07–2.80 (m, 5H, Ahx^ε, 3×F^β), 2.03 (t, 2H, $J_{\alpha,\beta}$ = 7.4 Hz, Ahx^α), 1.41 (p, 2H, $J_{\beta,\alpha}$ = $J_{\beta,\gamma}$ = 7.5 Hz, Ahx^β), 1.31 (m, 2H, Ahx^δ), 1.16 (m, 2H, Ahx^γ).

^{13}C{^{1}H} NMR (101 MHz, DMSO- d_6, 296 K, HSQC, HMBC) δ /ppm = 172.9 C=O^{Ahx}), 170.8 (C=O^{F3}), 170.6 (C=O^{F2}), 168.2 (C=O^{F1}), 138.2 ($F^{\gamma3}$), 137.9 ($F^{\gamma2}$), 135.3 ($F^{\gamma1}$), 130.2, 129.8, 129.7 (6×F^δ), 128.8, 128.5 (6×F^ε), 127.4, 126.7, 126.7 (3×F^ζ), 71.7, 70.9, 70.3, 70.2, 70.1, 70.0 (24×CH_2O), 68.8 (CH_2^{TRIS}), 60.2 (C_q^{TRIS}), 58.5 (OCH_3), 54.9, 54.8 (2×F^α), 53.7 ($F^{\alpha1}$), 38.9 (Ahx^ε), 38.5, 38.0, 37.2 (3×F^β), 36.3 (Ahx^α), 29.2 (Ahx^δ), 26.4 (Ahx^γ), 25.5 (Ahx^β).

Benzene-1,3,5-tri(*N*-[L-phenylalanyl-L-phenylalanyl-L-phenylalanyl-{ε-amino-*N*-[tris-*O*-(2,5,8,11,14-pentaoxa-pentadecyl)methyl]}hexanoylamidyl])carboxamide (**S8**)

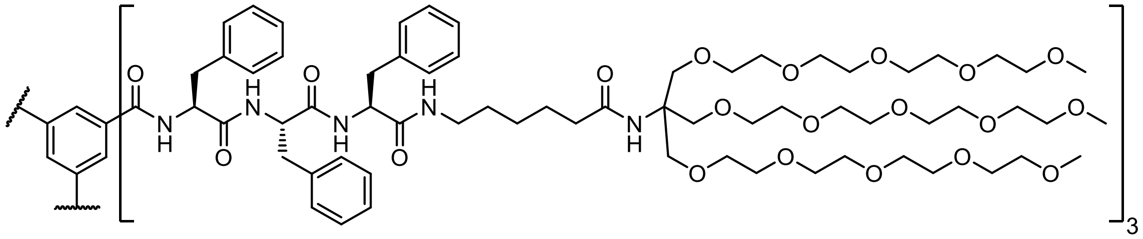

Trimesic acid (4.0 mg, 19 µmol, 1.0 equiv), PyBOP (38 mg, 74 µmol, 3.9 equiv), HOAt (10 mg, 74 µmol, 3.9 equiv) and $F_3AhxTRIS(EG_4OMe)_3$ (86 mg, 67 µmol, 3.5 equiv) were dissolved in 500 µL DMF. The resulting mixture was cooled to 0 °C and NMM (14.5 µL, 131 µmol, 6.9 equiv) was added in two portions. The reaction mixture was stirred at 0 °C for 15 min and subsequently warmed to room temperature. The stirring was continued for 15 h, the solvents removed in vacuo, and the residing oil subjected to SEC on S-X1 (DMF). After evaporation of the solvents the remaining film was hydrated and subjected to lyophilization.

Yield: 69 mg (17 µmol, 93%) colorless amorphous solid.

$C_{201}H_{309}N_{15}O_{60}$ (M = 3895.7280 g/mol, M_{mi} = 3893.1589 u).

TOF-MS (MALDI, pos., CHCA) m/z: 3916.164 $[M+Na]^+$ (calc. 3916.149).

^{1}H NMR (400 MHz, DMSO-d_6, 294 K, COSY, HSQC, HMBC) δ /ppm = 8.69 (m, 6H, N*H*), 8.24–8.18 (m, 3H, NH^F), 8.17 (s, 3H, CH^{BTA}), 7.83 (t, 3H, J_{NHCH2} = 5.6 Hz, NH^{Ahx}), 7.62 (s, 3H, NH^{TRIS}), 7.33–6.91 (m, 45H, $F^{\delta,\varepsilon,\zeta}$), 4.75 (m, 3H, F^α), 4.57 (m, 3H, F^α), 4.47 (m, 3H, F^α), 3.59 (sb, 18H, CH_2^{TRIS}), 3.52–3.45 (m, 126H, OCH_2^{EG}), 3.41 (m, 18H, OCH_2^{EG}), 3.22 (s, 27H, CH_3O), 3.11–2.75 (m, H, Ahx^ε{3.04/2.90}, F^β), 2.03 (m, 6H, Ahx^α), 1.41 (m, 6H, Ahx^β), 1.29 (m, 6H, Ahx^δ), 1.14 (m, 6H, Ahx^γ).

$BTAF_3AhxEG_2NH_2$, S9

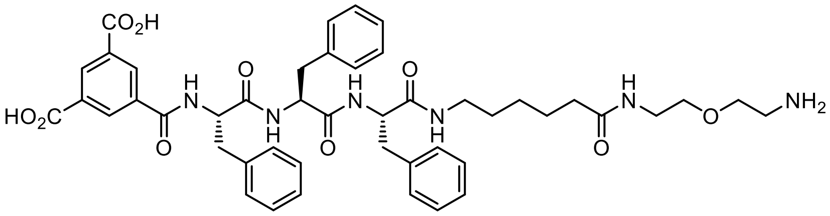

The polypeptide was synthesized using a custom solid-support from Rapp Polymere *O*-(aminoethyl)ethylene glycol TRT PS resin (0.16 mmol/g loading, 0.25 mmol scale) in a CS136XT synthesizer and according to a universal operating procedure:

For the automated coupling, the loaded dry resin was weighed into the reaction vessel of the synthesizer. All of the Fmoc-amino acids were provided as solutions in DMF, each in a dedicated reservoir.

Fmoc removal: The cleavage of the Fmoc-group of the terminal amino acid/peptide attached to the resin was achieved after treatment (2×) with a solution of piperidine in DMF (20 vol %) for 15 min.

Peptide coupling: Fmoc-amino acids (4.0 equiv) were preactivated in DMF with HBTU (2.9 mmol, 4.0 equiv), HOBt (3.2 mmol, 4.4 equiv), and DIPEA (4.3 mmol, 6.0 equiv) and this solution subsequently transferred to the reaction vessel and shaken for 1 h.

Each of these first two steps was followed by extensive washing steps, involving repeated DMF and DCM rinsing of the reaction vessel and the resin present in it. The final coupling of trimesic acid was performed manually after preactivation of the acid with HATU, HOAt, and DIPEA in NMP. After 20 min, the coupling solution was removed from the resin and the peptide was liberated with DCM/TFA/TIS 20:20:1 (3 × 5 mL), 30 min each. Codistillation against toluene was used to remove the reactants (3 × 15 mL).

Purification: RP HPLC (XBridge C18, 125 × 30, 3.5 µm, 130 Å, Waters, 10 → 100% B in 15 min, A: H_2O +0.1 vol % TFA, B: MeCN + 0.1vol % TFA).

Yield: 76 mg colorless lyophilizate.

$C_{46}H_{54}N_6O_{10}$ (M = 850.9700 g/mol, M_{mi} = 850.3901 u).

TOF-MS (ESI, pos.) m/z: 851.3974 $[M+H]^+$ (calc. 851.3974).

^{1}H NMR (600 MHz, DMSO-d_6, 294 K, HSQC, COSY) δ /ppm = 8.56–8.47 (m, 3H, CH^{BTA}), 7.34–7.04 (m, 15H, $F^{\delta,\varepsilon,\zeta}$), 4.70 (dd, 1H, $J_{\alpha,\beta a}$ = 4.2 Hz, $J_{\alpha,\beta b}$ = 10.9 Hz, F^α), 4.50 (dd, 1H, $J_{\alpha,\beta a}$ = 4.8 Hz, $J_{\alpha,\beta b}$ = 9.0 Hz, F^α), 4.43 (dd, 1H, $J_{\alpha,\beta a}$ = 6.0 Hz, $J_{\alpha,\beta b}$ = 8.9 Hz, F^α), 3.56 (d, 2H, $J_{CH2,CH2}$ = 5.1 Hz, CH_2O^{EG}), 3.41 (d, 2H, $J_{CH2,CH2}$ = 5.7 Hz, CH_2O^{EG}), 3.21 (d, 2H, $J_{CH2,CH2}$ = 5.6 Hz, CH_2O^{EG}), 3.05–2.74 (m, 9H, Ahx^ε, 3×F^β, CH_2NH_2EG), 2.07 (t, 2H, $J_{\alpha,\beta}$ = 7.5 Hz, Ahx^α), 1.43 (p, 2H, $J_{\beta,\alpha}$ = $J_{\beta,\gamma}$ = 7.6 Hz, Ahx^β), 1.29 (m, 2H, Ahx^δ), 1.13 (m, 2H, Ahx^γ).

BTAF$_3$AhxEG$_2$AcN$_3$, S10

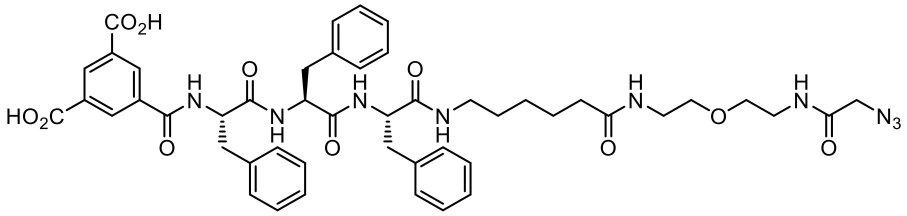

Peptide **S9** (20 mg, 22.5 µmol, 1.0 equiv) was dissolved in 0.5 mL DMF and N_3AcNHS (4.9 mg, 24.8 µmol, 1.1 equuiv) was added in two portions at room temperature, followed by the subsequent addition of NMM (7.7 µL, 70.0 µmol, 3.1 equiv). The reaction mixture was stirred for 4 h, neutralized with TFA, and purified by SEC on LH-20 (DMF).

Yield: 17 mg (18.2 µmol, 81%) colorless lyophilizate.

$C_{48}H_{55}N_9O_{11}$ (M = 934.0200 g/mol, M_{mi} = 933.4021 u).

TOF-MS (ESI, neg.) m/z: 932.3948 $[M-H]^-$ (calc. 932.3948).

^{1}H NMR (600 MHz, DMSO-d_6, 296 K) δ /ppm = 8.55 (sb, 3H, CH^{BTA}), 7.33–7.04 (m, 15H, $F_{\delta,\varepsilon,\zeta}$), 4.73 (ddd, 1H, $J_{\alpha,\beta a}$ = 4.1 Hz, $J_{\alpha,NH}$ = 8.6 Hz, $J_{\alpha,\beta b}$ = 9.4 Hz, F^α), 4.54 (dd, 1H, $J_{\alpha,\beta a}$ = 4.8 Hz, $J_{\alpha,\beta b}$ = 8.3 Hz, F^α), 4.46 (dd, 1H, $J_{\alpha,\beta a}$ = 6.0 Hz, $J_{\alpha,\beta b}$ = 8.1 Hz, F^α), 3.82 (s, 2H, CH_2N_3), 3.39 (m, 4H, 2×CH_2O^{EG}), 3.24 (m, 2H, CH_2O^{EG}), 3.18 (m, 2H, CH_2O^{EG}), 3.07–2.70 (m, 8H, Ahx$^\varepsilon$, 3×Fβ, C$H_2NH_2^{EG}$), 2.04 (t, 2H, $J_{\alpha,\beta}$ = 7.5 Hz, Ahx$^\alpha$), 1.44 (m, 2H, Ahx$^\beta$), 1.29 (m, 2H, Ahx$^\delta$), 1.14 (m, 2H, Ahx$^\gamma$).

S11

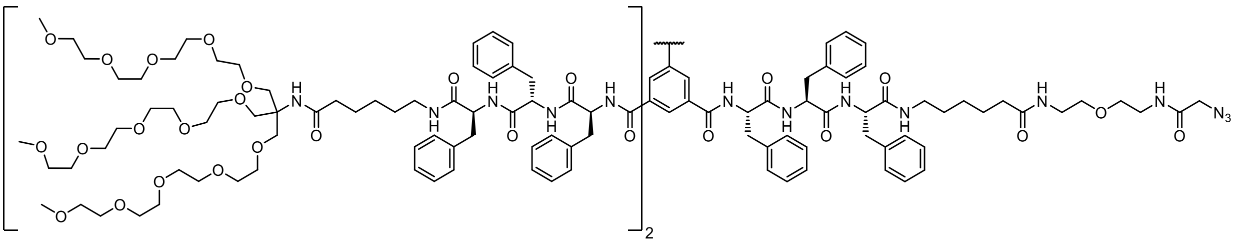

Amphiphile F$_3$AhxTRIS(EG$_4$OMe)$_3$ (48 mg, 37 µmol, 2.5 equiv) was dissolved in 1 mL DMF and subsequently added to a solution of diacid **S10** (14 mg, 15 µmol, 1.0 equiv), PyBOP (20 mg, 37 µmol, 2.5 equiv), and HOAt (4 mg, 30 µmol, 2.0 equiv) in 1 mL DMF. Then, NMM (10 µL, 90 µmol, 6.0 equiv) was added in small portions and the stirring was continued at room temperature for 18 h. The reaction mixture was diluted with 5 mL MeOH and the solvents subsequently removed in vacuo. The remaining crude product was purified by SEC on S-X1 (DMF).

Yield: 48 mg (14.2 µmol, 94%) colorless lyophilizate.

$C_{176}H_{257}N_{19}O_{47}$ (M = 3391.0780 g/mol, M_{mi} = 3388.8304 u).

TOF-MS (ESI, pos.) m/z: 1717.4092 $[M+2Na]^{2+}$ (calc. 1717.4044).

^{1}H NMR (600 MHz, DMSO-d_6, 296 K, COSY, HSQC, HMBC) δ /ppm = 8.18 (sb, 3H, CH^{BTA}), 7.39–6.95 (m, 45H, $F^{\delta,\varepsilon,\zeta}$), 4.74 (m, 3H, 3×$F^{\alpha}$), 4.57 (m, 3H, 3×$F^{\alpha}$), 4.47 (m, 3H, 3×$F^{\alpha}$), 3.81 (s, 2H, C$H_2N_3$), 3.59 (sb, 12H, 6×C$H_2^{TRIS}$), 3.54–3.46 (m, 96H, 48×CH_2O^{EG}), 3.41 (m, 4H, 2×CH_2O^{EG}), 3.24 (m, 2H, CH_2O^{EG}), 3.27–3.14 (m, 24H, 6×CH_3O^{EG} {3.22}, 3×CH_2O^{EG}), 3.08–2.70 (m, 26H, 3×Ahx$^{\varepsilon}$, 9×F^{β}, CH_2NH^{EG}), 2.03 (td, 6H, $J_{\alpha,\beta}$ = 7.5 Hz, $J_{\alpha,NH}$ = 4.3 Hz, Ahx$^{\alpha}$), 1.42 (m, 6H, Ahx$^{\beta}$), 1.28 (m, 6H, Ahx$^{\delta}$), 1.14 (m, 2H, Ahx$^{\gamma}$).

S12

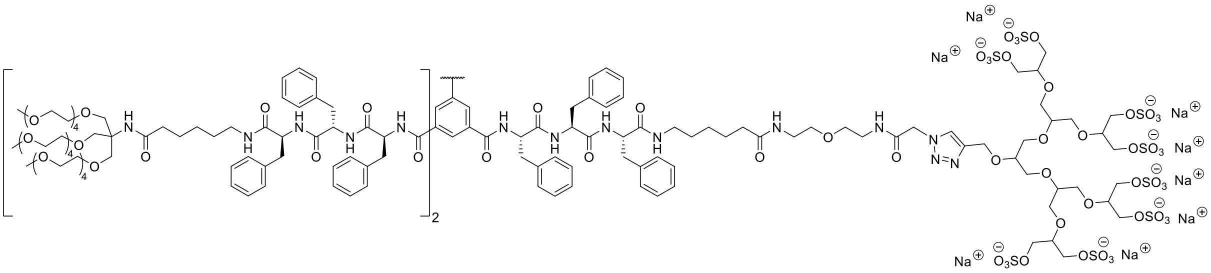

Freshly prepared stock solutions of the reactants were prepared prior to the reaction. Following concentrations were provided:

A S12 20 mg/mL in DMF
B Propargylated dendron 20 mg/mL in DMF
C TBTA 25 mM in DMF
D $CuSO_4 \times 5H_2O$ 50 mM in H_2O
E NaAsc 100 mM in H_2O

Solutions A (2.0 µmol, 1 equiv), B (2.2 equiv), C (1.6 equiv), and D (2.0 equiv) were dispensed and diluted with a mixture of TFE/DMF 1:1 to reach a final concentration of 1 mg/mL, calculated for the functional monomer. The mixture was purged with argon and subjected to two freeze-pump-thaw cycles. Subsequently, solution E (4 equiv) was introduced and the reaction mixture was once more subjected to a freeze-pump-thaw cycle. The reaction proceeded over 3 d at 50 °C. The solvents were removed in vacuo and the residing product taken up in DMF and subjected to SEC on S-X1 (DMF). The DMF was removed in vacuo and the residing film rehydrated with H_2O to yield the corresponding conjugate after lyophilization.

Yield: 6 mg (1.7 µmol, 85%) colorless amorphous lyophilizate.

$C_{200}H_{295}N_{19}Na_8O_{86}S_8$ (M = 4782.0052 g/mol, M_{mi} = 4778.6242 u).

^{1}H-^{13}C HSQC (600 MHz/151 MHz, DMSO-d_6, 294 K) only selected signals, δ /ppm = 8.16/129.5 (CH_{BTA}), 8.06/125.9 ($CH_{triazole}$).

^{1}H-^{13}C HMBC (600 MHz/151 MHz, DMSO-d_6, 294 K) only selected signals, δ /ppm = 8.16/165.7 (C=O/H-2,4,6_{BTA}), 8.16/129.5 (C/H-2,4,6_{BTA}), 8.06/144.7 (C-4/H-$5_{triazole}$).

4 Spectra

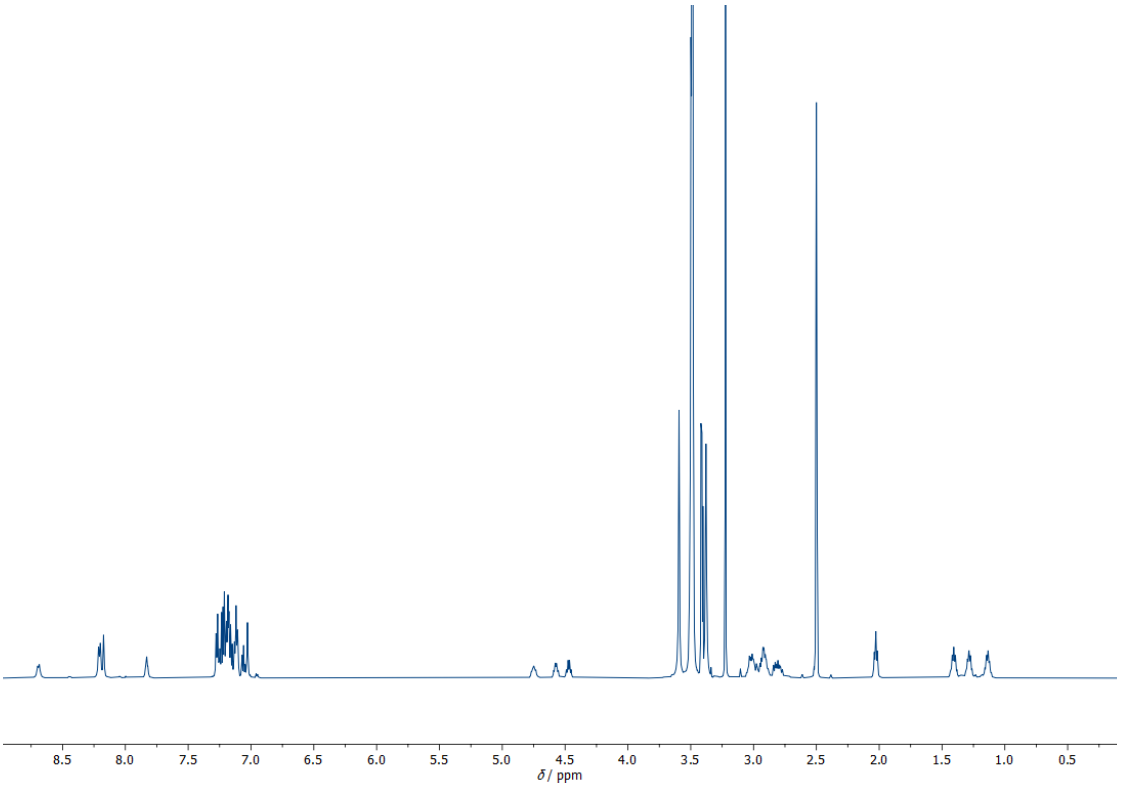

Figure S5: ^{1}H NMR spectrum of **I** (600 MHz, DMSO-d_6, 294 K).

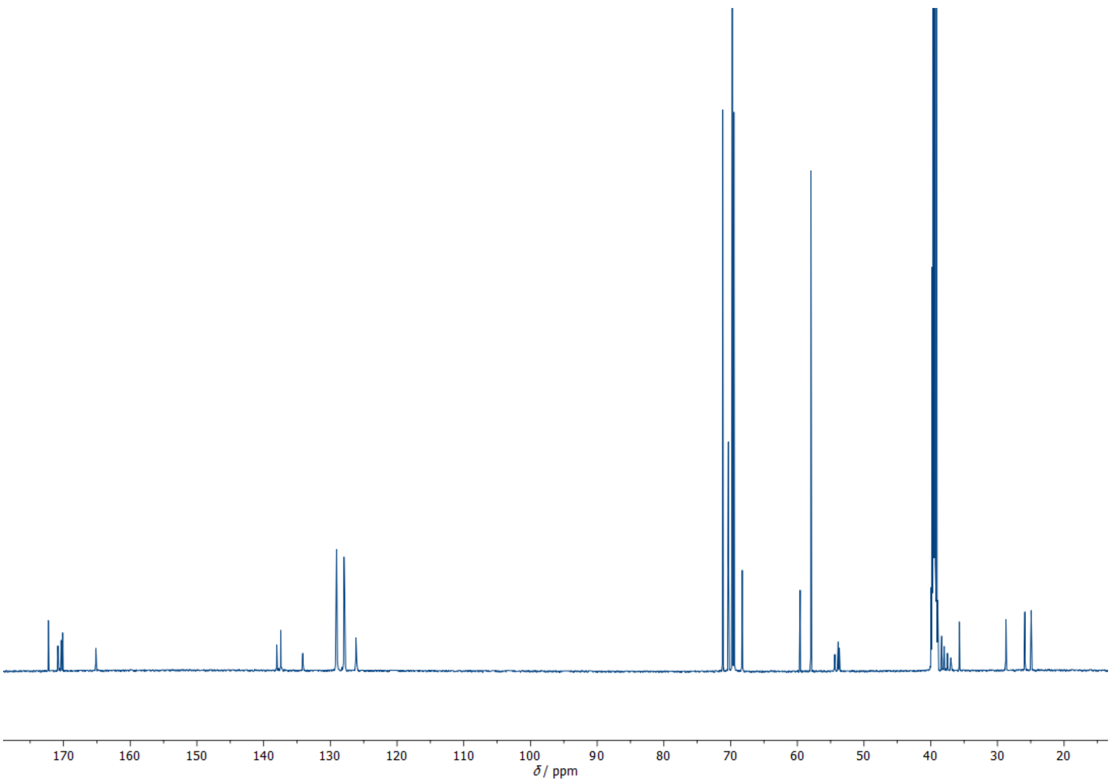

Figure S6: ^{13}C NMR spectrum of **I** (151 MHz, DMSO-d_6, 294 K).

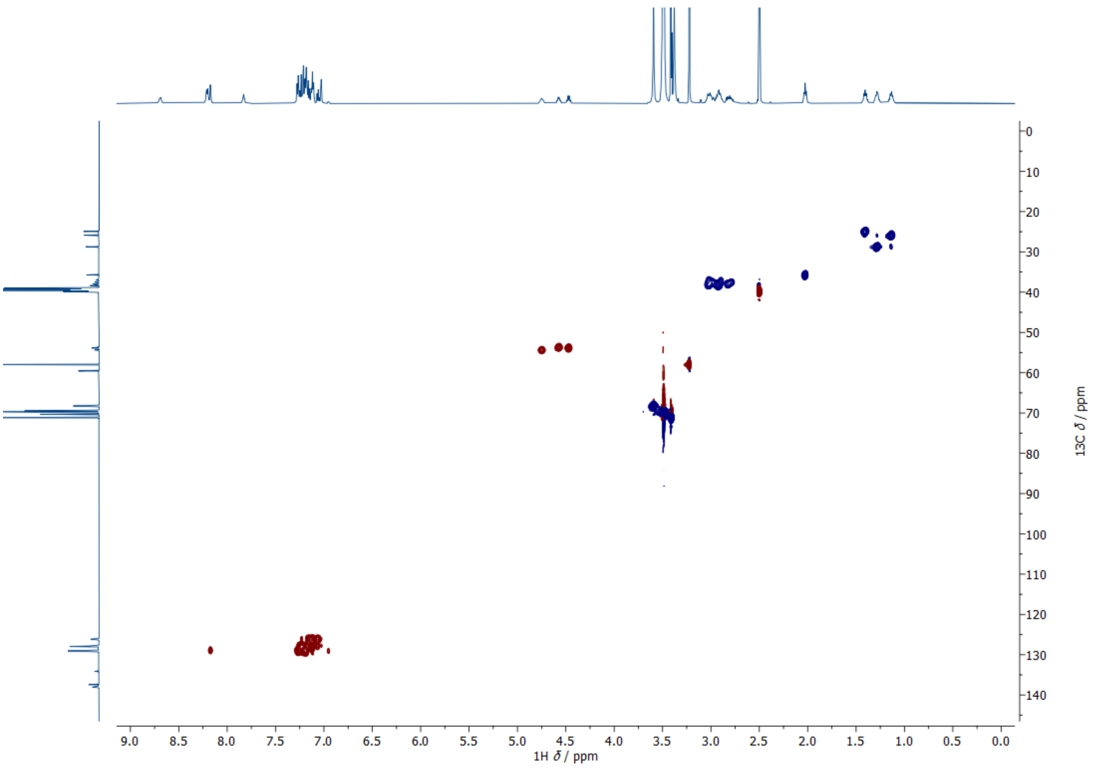

Figure S7: HSQC NMR spectrum of **I** (600 MHz, DMSO-d_6, 294 K).

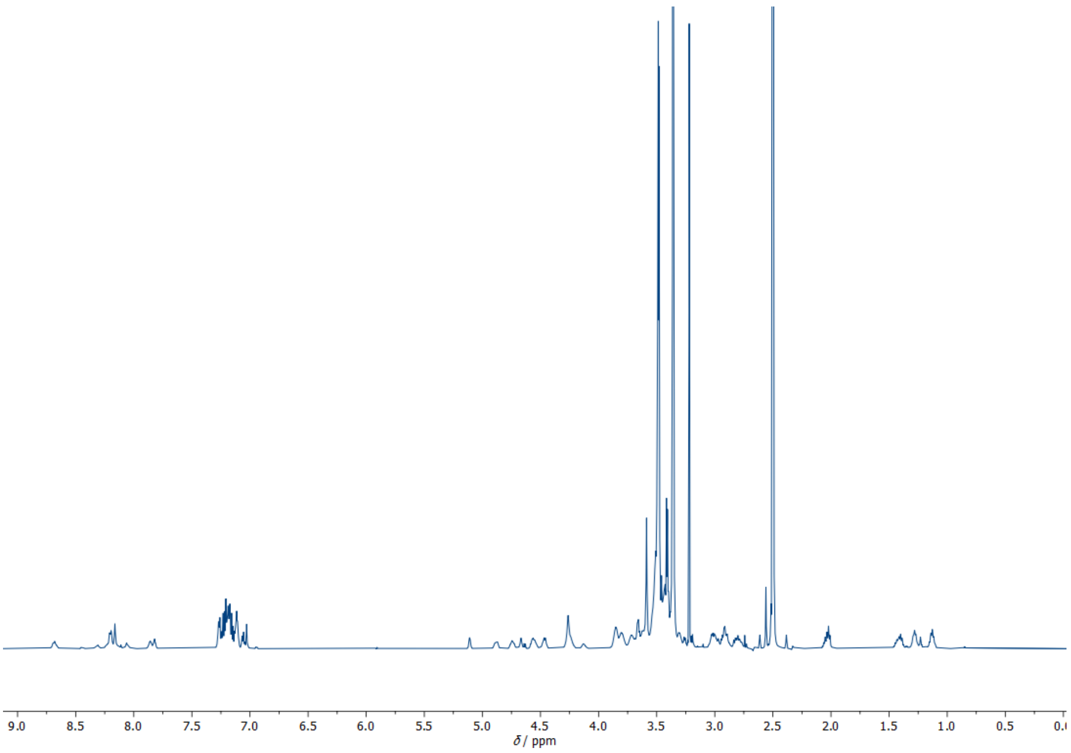

Figure S8: ^{1}H NMR spectrum of **II** (600 MHz, DMSO-*d*$_6$, 294 K).

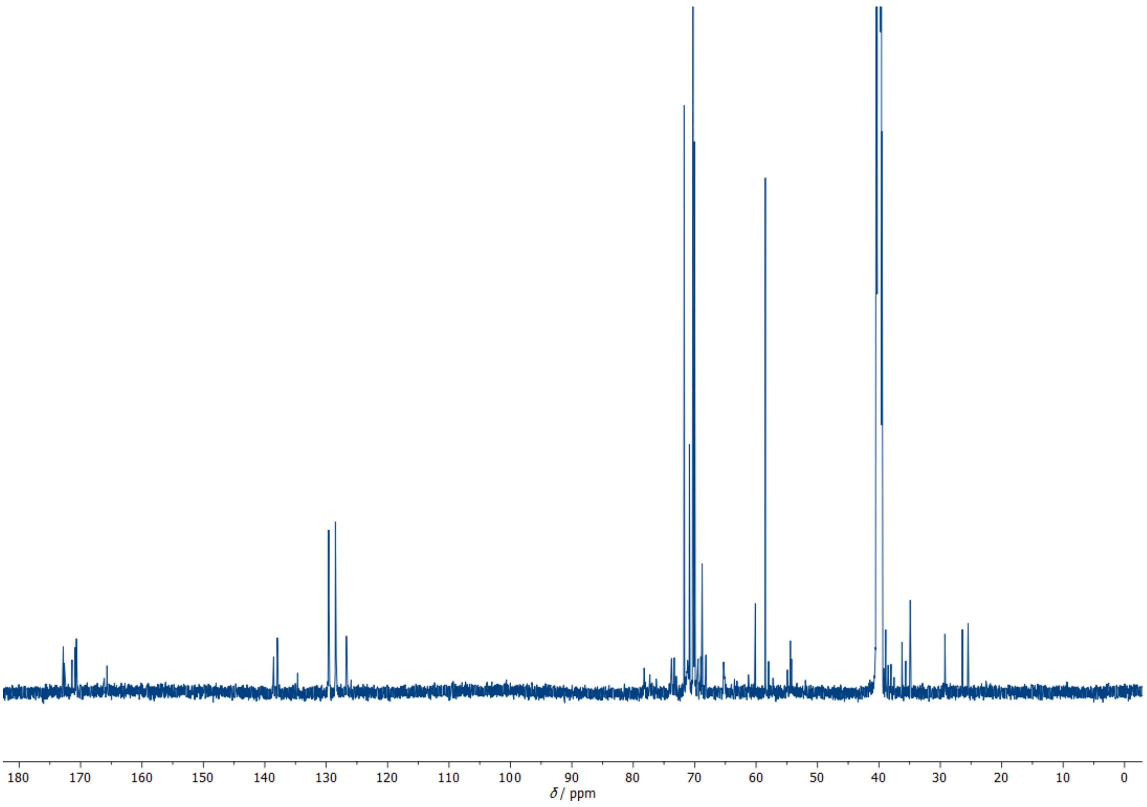

Figure S9: ^{13}C NMR spectrum of **II** (151 MHz, DMSO-*d*$_6$, 294 K).

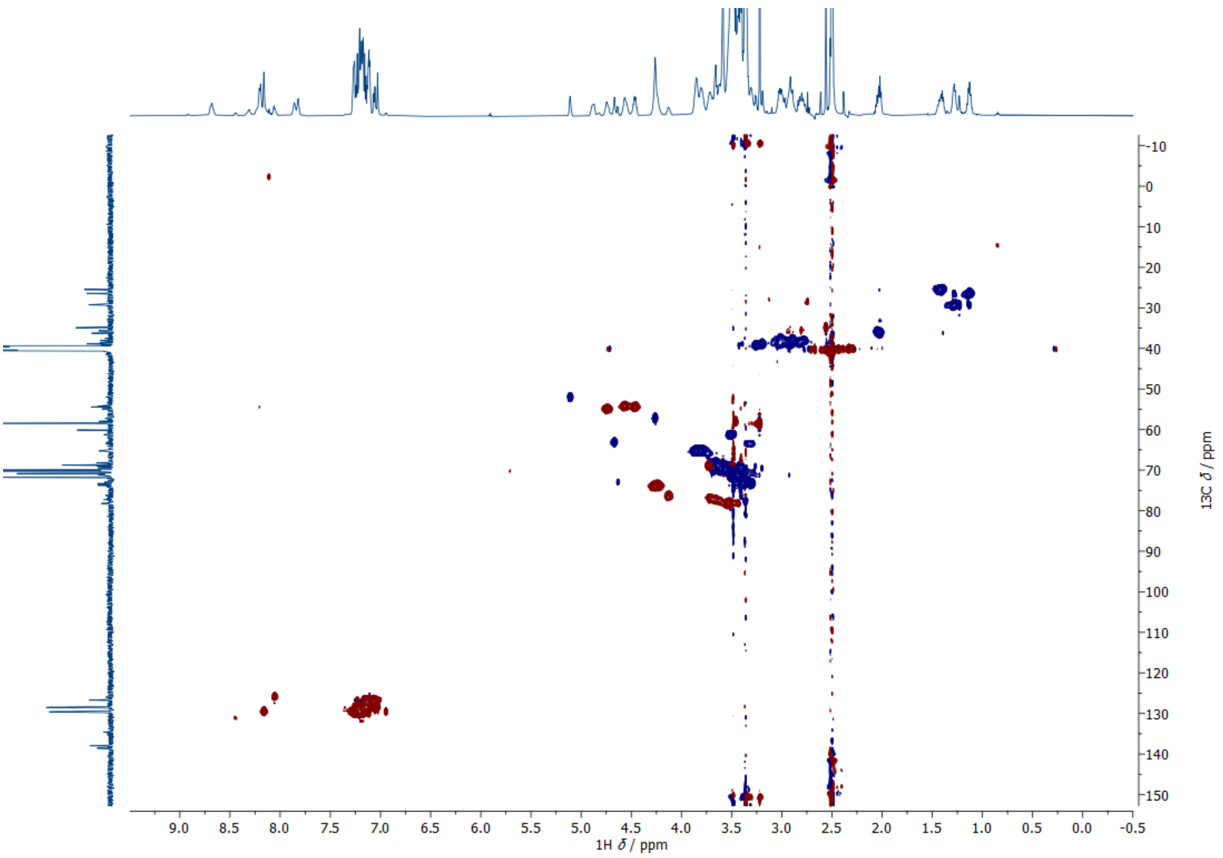

Figure S10: HSQC-NMR spectrum of **II** (600 MHz, DMSO-d_6, 294 K).

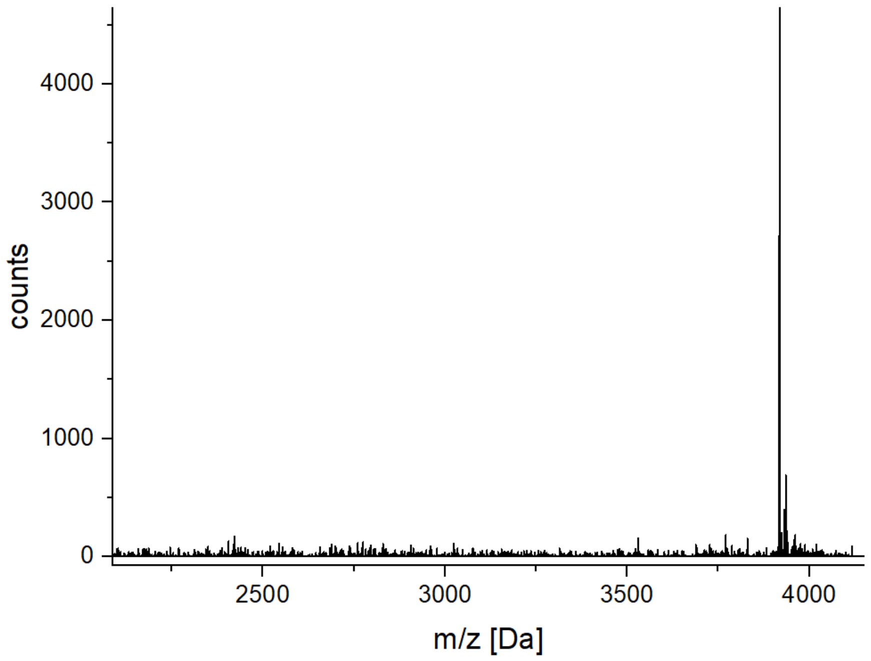

Figure S11: MALDI spectrum of **I** (CHCA).

5 References

[S1] W. L. Armarego, D. Perrin, *Purification of Laboratory Chemicals*, 1997.

[S2] S. Ehrmann, C. W. Chu, S. Kumari, K. Silberreis, C. Böttcher, J. Dernedde, B. J. Ravoo, R. Haag, *J. Mater. Chem. B* **2018**, *6* , 4216.

[S3] I. Donskyi, M. Drüke, K. Silberreis, D. Lauster, K. Ludwig, C. Kühne, W. Unger, C. Böttcher, A. Herrmann, J. Dernedde, M. Adeli, R. Haag, *Small* **2018**, 14, 1800189.

[S4] J. Dernedde, A. Rausch, M. Weinhart, S. Enders, R. Tauber, K. Licha, M. Schirner, U. Zugel, A. von Bonin, R. Haag, *Proc. Natl. Acad. Sci. USA* **2010**, *107*, 19679.

[S5] J. Vonnemann, S. Liese, C. Kuehne, K. Ludwig, J. Dernedde, C. Böttcher, R. Netz, R. Haag, *J. Am. Chem. Soc.* **2015**, *137*, 2572.

[S6] I. Papp, J. Dernedde, S. Enders, R. Haag, *Chem. Commun.* **2008**, *44*, 5851.
